# Portal dosimetry in wedged beams

**DOI:** 10.1120/jacmp.v16i3.5375

**Published:** 2015-05-08

**Authors:** Hanno Spreeuw, Roel Rozendaal, Priscilla Camargo, Anton Mans, Markus Wendling, Igor Olaciregui‐Ruiz, Jan‐Jakob Sonke, Marcel Van Herk, Ben Mijnheer

**Affiliations:** ^1^ Department of Radiation Oncology The Netherlands Cancer Institute Amsterdam The Netherlands; ^2^ Centro de Metrologia das Radiações Instituto de Pesquisas Energéticas e Nucleares (IPEN/CNEN) São Paulo Brazil; ^3^ Department of Radiation Oncology Radboud University Medical Center Nijmegen The Netherlands

**Keywords:** EPID, portal dosimetry, treatment verification, *in vivo* dosimetry, wedged beams

## Abstract

Portal dosimetry using electronic portal imaging devices (EPIDs) is often applied to verify high‐energy photon beam treatments. Due to the change in photon energy spectrum, the resulting dose values are, however, not very accurate in the case of wedged beams if the pixel‐to‐dose conversion for the situation without wedge is used. A possible solution would be to consider a wedged beam as another photon beam quality requiring separate beam modeling of the dose calculation algorithm. The aim of this study was to investigate a more practical solution: to make aSi EPID‐based dosimetry models also applicable for wedged beams without an extra commissioning effort of the parameters of the model. For this purpose two energy‐dependent wedge multiplication factors have been introduced to be applied for portal images taken with and without a patient/phantom in the beam. These wedge multiplication factors were derived from EPID and ionization chamber measurements at the EPID level for wedged and nonwedged beams, both with and without a polystyrene slab phantom in the beam. This method was verified for an EPID dosimetry model used for wedged beams at three photon beam energies (6, 10, and 18 MV) by comparing dose values reconstructed in a phantom with data provided by a treatment planning system (TPS), as a function of field size, depth, and off‐axis distance. Generally good agreement, within 2%, was observed for depths between dose maximum and 15 cm. Applying the new model to EPID dose measurements performed during ten breast cancer patient treatments with wedged 6 MV photon beams showed that the average isocenter underdosage of 5.3% was reduced to 0.4%. Gamma‐evaluation (global 3%/3 mm) of these *in vivo* data showed an increase in percentage of points with γ≤1 from 60.2% to 87.4%, while $\gamma_{\text{mean}}$ reduced from 1.01 to 0.55. It can be concluded that, for wedged beams, the multiplication of EPID pixel values with an energy‐dependent correction factor provides good agreement between dose values determined by an EPID and a TPS, indicating the usefulness of such a practical solution.

PACS numbers: 87.55.km, 87.55.kd, 87.55.Qr, 87.56a.ng

## I. INTRODUCTION

Electronic portal imaging devices (EPIDs) are widely used for patient positioning. As a result of their favorable characteristics, such as a stable dose‐response relationship and immediate availability of data in digital format, some centers have also chosen EPIDs as their main tool for pretreatment or *in vivo* dose verification.^(1)^ The number of groups and institutions that use EPID dosimetry clinically is increasing and there is a high potential for EPIDs to become a routine tool for *in vivo* dose verification.^(2)^

Wedges are important beam modifiers utilized in radiotherapy and widely applied routinely, and several groups have investigated EPID dosimetry in wedged beams. These studies concerned pretreatment verification of wedged beams by means of portal dosimetry at the EPID level,^3^, ^4^, ^5^ verification of the exit dose,^(6)^ determination of the midplane dose at a point^7^, ^8^ or the midplane 2D dose distribution,^9^, ^10^ and the use of EPID dosimetry to determine wedge factors.^10^, ^11^ Different types of EPIDs, as well as different types of correction procedures, were used in these studies. In the model used by Boellaard et al.,^6^, ^9^ which was applied in combination with a liquid‐filled matrix ionization chamber EPID, no correction was needed for wedged beams. The models that predict CCD camera‐based EPID dose images^3^, ^4^, ^5^ required a correction for the change in EPID response due to beam hardening introduced by the insertion of a wedge in an open beam. For this purpose, Pasma et al.^(4)^ incorporated two functions in their nonwedged beam model to take the effects resulting from placing wedges in the beam into account. Nijsten et al.^(5)^ introduced in their model an extra factor for wedged fields to correct for the nonlinear response of camera‐based EPIDs to a different photon energy spectrum. Renner et al.^(10)^ and Budgell et al.^(11)^ noted that wedge factors determined with an amorphous silicon (aSi) EPID did not agree with those measured with an ionization chamber. Because the aim of the work of Budgell and colleagues was to detect only the variation in wedge factors, they did not correct the EPID response in wedged beams. However, Renner et al., when observing that their open‐beam algorithm was unable to accurately reproduce the dose from wedged fields for aSi EPIDs, proposed to use a heavily filtered beam to determine the parameters required in their model. A similar reduction in EPID response was observed by Kirkby and Sloboda^(12)^ when introducing a steel compensator in a 6 MV photon beam. These authors proposed to position a 7 mm thick copper plate in front of the aSi EPID to reduce the uncertainty in the EPID response when a thick absorber is placed in a photon beam. Fidanzio et al.^(7)^ implemented a method for *in vivo* dosimetry of breast tangential irradiation using wedged beams. In that work, the transit signal along the central beam axis of 150 and 300 wedged beams was measured with an aSi EPID and correlated to the dose at the breast midpoint. Phantom measurements showed that this ratio was within the experimental uncertainty of ±0.5%, the same for the two wedge angles investigated in their study. More recently, the method developed by this group was generalized and extended to wedged beams supplied by linacs of different manufacturers.^(8)^

These solutions concern corrections to be applied in wedged beams specific for liquid‐filled or CCD camera‐based types of EPIDs, require elaborate calibration methods for wedged beams when using aSi EPIDs, or provide the dose only at a single point in the patient. Several groups have developed 2D dosimetry methods using aSi EPIDs for pretreatment verification of open‐beam treatments (i.e., without a wedge and phantom in the beam).^13^, ^14^ Other groups have developed transit dosimetry models for aSi EPIDs (i.e., for treatments with a patient/phantom in the beam).^15^, ^16^, ^17^, ^18^ Calibration of the EPID in these models is generally performed by converting EPID pixel values to dose values using ionization chamber measurements. When introducing a wedge in the beam, the fraction of low‐energy photons in the beam will change and, therefore, will alter the EPID response compared to the situation without wedge. This change in energy spectrum is a complicated function of the wedge thickness (i.e., of the off‐axis position of a specific pixel in the EPID). Correcting for this change in conversion of pixel‐to‐dose value can be done by applying a sophisticated beam calibration model, as for instance proposed by Nijsten et al.^(19)^ These authors developed a correction model for aSi EPIDs for differences in energy spectrum, which was dependent on off‐axis position and patient/phantom thickness. Such a modification would require, however, a large number of additional measurements for wedged beams. A much easier solution would be to make some simple adjustments for wedged beams to an existing open‐beam dosimetry model without an extra commissioning effort of the parameters of such a model. The aim of this study was to investigate the accuracy and clinical usefulness of such a more practical solution.

## II. MATERIALS AND METHODS

### A. EPID dosimetry in beams without wedges

aSi EPIDs (Perkin Elmer RID 1680 AL5 (PerkinElmer, Waltham, MA) /Elekta iViewGT Elekta, Crawley, UK)) mounted on SL20i linear accelerators (Elekta) are utilized for all dose verification measurements. Photon beam energies of 6, 10, and 18 MV were used in this study. The linacs have a motorized internal wedge with a 60° fixed angle. The wedge material is cast lead/antimony alloy (96% lead and 4% antimony) with a mass density of $11.1\;g/{\text{cm}}^{3}$. The EPIDs are located at 160 cm distance from the linac focus and have in our institution an additional 2.5 mm thick copper plate to absorb low‐energy photons scattered from the phantom or patient.^15^, ^20^ Images are acquired using in‐house developed software and resampled at 256×256 pixels resulting in an effective pixel size of 1 mm^2^ in the isocenter plane.^20^, ^21^ The average lifetime of the aSi panels, which are intensively used in our institution, is currently about 32 months, and is increasing since we stopped using 18 MV photons.

The complete description and assumptions made for our nonwedged beam algorithm for 2D and 3D dose reconstruction were presented by Wendling et al.^15^, ^16^ In short, the EPID‐based, back‐projection dose reconstruction model takes into account primary dose and a scatter contribution. The latter is computed by convolving a fraction of the former with a single water‐based kernel. As a consequence, our dose reconstruction model is not applicable for regions with tissue heterogeneities, as discussed more extensively by Wendling et al.^15^, ^16^ This fraction, the normalized scatter‐to‐primary ratio, is dependent on both depth and lateral position. The primary dose in a patient/phantom derived from a certain pixel, *ij*, in the EPID, ${\mathrm{Pr}}_{\mathrm{ij}}$, at a point at a distance $d_{\mathrm{reconst}}$ of the reconstruction plane from the accelerator target is given by:

(1) $${\mathrm{Pr}}_{ij}(d_{reconst})={\mathrm{Pr}}_{ij}^{EPID}\cdot {\left(\frac{d_{reconst}}{d_{EPID}}\right)}^{-2}\cdot AC_{ij}(d_{reconst})$$

where $Pr_{ij}^{EPID}$ is the primary dose at the level of the EPID, $d_{\mathrm{EPID}}$ is the distance of the EPID from the accelerator target, and $(d_{reconst}/{d_{EPID})}^{-2}$ is the distance correction determined by the inverse‐square law. The attenuation between the reconstruction plane and the exit surface of the patient/phantom is given by the attenuation correction, ${\mathrm{AC}}_{\mathrm{ij}}$, and can be derived from the radiological path length from the entrance surface to the reconstruction plane, $d_{\mathrm{ij}}^{\mathrm{radiol}}$, as follows:

(2) $$AC_{ij}(d_{reconst})=T^{primary}(d_{ij}^{radiol})/T^{primary}$$

where $T^{\text{primary}}(d_{\text{ij}}^{\text{radiol}})$ is the transmission of the primary dose at $d_{\mathrm{ij}}^{\mathrm{radiol}}$ and $T^{\mathrm{primary}}$ the transmission of the primary dose through the patient/phantom, which is experimentally determined by dividing the primary portal dose image $PD_{ij}^{EPID}$ behind the patient/phantom by the corresponding “open image” (without the patient/phantom):

(3) $$T^{primary}=PD_{ij}^{EPID,\;with\;patient}/PD_{ij}^{EPID,\;without\;patient}$$

The primary portal dose image behind the patient is measured during the actual patient treatment, while the corresponding open image is measured either just before or after the patient treatment.

The attenuation correction in an arbitrary plane parallel to the EPID is now obtained by adding the exponent $d_{\text{ij}}^{\text{radiol}}/t_{\text{ij}}^{\text{radiol}}$ to $T^{\text{primary}}$, in which $t_{\text{ij}}^{\text{radiol}}$ is the radiological thickness of the patient determined at pixel ij. Details of this procedure can be found elsewhere.^15^, ^16^ When introducing a wedge in the beam, it is assumed in our new model that, when determining the attenuation correction, only $T^{\text{primary}}$ has to be corrected because the exponent has the same value as for the situation without a wedge.

If the dose is reconstructed in the radiological midplane, then the attenuation correction can in first approximation be written as $1/\surd T^{\text{primary}}$. The primary dose at the midplane is proportional to ${\text{Pr}}^{\text{EPID}}/\surd T^{\text{primary}}$. When using wedged beams in combination with the calibration of the EPID for nonwedged beams, the determination of both ${\text{Pr}}^{\text{EPID}}$ and $T^{\text{primary}}$ will be modified due to the change in photon energy spectrum by the insertion of a wedge in the beam.

### B. EPID dosimetry in beams with wedges

In order to take the change in photon energy spectrum caused by the insertion of a wedge in the beam into account, the set of EPID calibration parameters used in our back‐projection model has to be determined for wedged beams, which would require additional measurements. The method we propose in this study is to apply only two correction factors per photon energy, one for the portal image with a patient/phantom in the beam and one for the portal image without a patient/phantom in the beam, to correct for the effect of the wedge on the EPID dose reconstruction, while still using the calibration for nonwedged beams.

The two wedge multiplication factors, $\text{wm}f^{\text{air}}$ and $\text{wm}f^{\text{ph}}$, are derived for the three beam energies from the ratio of the ionization chamber (IC) dose value and the corresponding EPID dose value with and without a wedge in the beam, indicated with the subscript w and nw, respectively. For this purpose a Semiflex ionization chamber (SDD=160 cm) was embedded in a PMMA miniphantom (3 cm diameter for 6 and 10 MV, and 4 cm diameter for 18 MV, and PMMA buildup of 1.5, 2.6, and 3 cm for 6, 10, and 18 MV, respectively), at the position of the EPID (SDD=160 cm) at the center of a $10\times 10\;{\text{cm}}^{2}$ field. The measurements were performed for two collimator angles (90° and 270°). The resulting wm*f* values are now given by:

(4) $$wmf^{air}=\frac{D_{w}^{air}(IC)/D_{w}^{air}(EPID)}{D_{nw}^{air}(IC)/D_{nw}^{air}(EPID)}$$

(5) $$wmf^{ph}=\frac{D_{w}^{ph}(IC)/D_{w}^{ph}(EPID)}{D_{nw}^{ph}(IC)/D_{nw}^{ph}(EPID)}$$

For all phantom irradiations we used the same 20 cm thick (and 30 cm×30 cm wide) polystyrene slab phantom. Data were acquired on two accelerators.

Elekta accelerators have only one wedge with a 60° wedge angle. Beams with reduced wedge angle have contributions with and without this 60° wedge. This is not a problem if the separate EPID images from the wedged and nonwedged beam irradiations are available, because then only the wedged beam image has to be corrected. However, if only the sum of the two EPID images can be analyzed, then a straightforward application of our new method is not possible. In order to obtain the correct composite dose for such a situation, the total dose is first reconstructed from the original total portal image and then multiplied with an appropriate correction factor to derive the contribution of the fraction with the wedge in the beam. For this purpose, a wedge correction factor, wcf, was introduced, defined as the ratio between the EPID‐based reconstructed dose at 10 cm depth in a 20 cm thick polystyrene slab phantom, with and without wm*f* corrections to the portal images for a $10\times 10\;{\text{cm}}^{2}\;60^{\circ }$ wedged field. Values for wc*f* were derived by averaging over the volume of a sphere with a radius of 0.5 cm centered at the isocenter in the phantom. For irradiations with reduced wedge angle a partial wedge correction factor, $\text{wc}f_{\text{partial}}$, then has to be applied, given by:

(6) $$wcf_{partial}=\frac{wf\cdot MU_{wedge}\cdot wcf+MU_{nowedge}}{wf\cdot MU_{wedge}+MU_{nowedge}}$$

in which ${\mathrm{MU}}_{\mathrm{wedge}}$ and ${\mathrm{MU}}_{\mathrm{nowedge}}$ are the number of wedged and nonwedged beam monitor units, respectively; *wf* is the wedge factor, which takes the attenuation of the beam by the wedge into account. w*f* is defined as the dose at 10 cm depth inside a phantom for a $10\times 10\;{\text{cm}}^{2}$ field for a wedged beam relative to the dose for a nonwedged beam, for the same number of monitor units. Values for w*f* are available from ionization chamber measurements used for commissioning of the TPS. There is a small dependence of w*f* on photon beam energy, and its value varies between 0.276 and 0.299.

The midplane dose is proportional to ${\text{Pr}}^{\text{EPID}}/\surd T^{\text{primary}}$ Therefore, when inserting a wedge in the beam, ${\text{Pr}}^{\text{EPID}}$ has to be corrected by multiplying with $\text{wm}f^{\text{ph}}$, while $T^{\text{primary}}$ has to be multiplied with $\text{wm}f^{\text{ph}}$ / $\text{wm}f^{\text{air}}$ yielding an approximate value for the wedge correction factor for the total midplane dose:

(7) $$wcf∼\surd wmf^{ph}\cdot \surd wmf^{air}$$

Note that the wedge multiplication factors, *wmf*, are introduced to correct the EPID images for a 60° wedge in the beam, while the wedge correction factors, *wcf*, are needed in case of irradiations with beams with reduced wedge angle and the separate images of the open and wedged beam are not available.

### C. Verification of the dose reconstruction by comparison with predictions from the TPS

All dose reconstructions were compared with the predictions from the clinically used TPS (Pinnacle 9.2, Philips Medical Systems, Eindhoven, The Netherlands). The TPS dose values were calculated using a grid with a voxel size of 2 mm in all dimensions, while the EPID pixel pitch of 1 mm in the midplane was downsampled to 2 mm. Previous measurements have shown that Pinnacle dose values along the central beam axis of wedged beams agree within approximately 1% at depths beyond dose maximum along the central beam axis, and between about 1% and 2% off‐axis in the high‐dose region (data not shown).

### D. Phantom dose verifications

The complete description and assumptions made for our nonwedged beam algorithm for 2D and 3D dose reconstruction are presented by Wendling et al.^15^, ^16^ In these papers it also has been described that our method has been checked extensively for many situations including various off‐axis distances and different isocenter positions with respect to the patient geometry. The limitations of our new wedged beam approach were assessed by comparing reconstructed dose values with planned dose values at and away from the isocenter — along the central beam axis and in the lateral plane through the isocenter. In order to assess the field size dependence of wm*f* we used a set of field sizes ranging from 3×3 to $23\times 23\;{\text{cm}}^{2}$ to irradiate the isocentrically aligned polystyrene slab phantom for all three energies. In addition, image acquisition of the same fields without a phantom in the beam was performed. The average dose in the phantom within a sphere with a radius of 0.5 cm from the isocenter as predicted by our TPS was compared with the average dose reconstructed from EPID measurements after converting the portal images taken with and without the phantom in the beam with the appropriate wm*f* values. For the on‐axis comparison, depth dose curves for a $10\times 10\;{\text{cm}}^{2}$ field size of a wedged beam were derived from the TPS and the EPID reconstructions and compared. Because the effect of inserting a wedge in the beam is largest at 6 MV, this comparison was only done at this energy. Similarly, for the off‐axis region beam profiles from the EPID‐based reconstructed dose distribution at 10 cm depth were compared with planned profiles, both in the direction with and without a wedge gradient. It should be noted that additional measurements using different phantom thicknesses may be useful in order to confirm that for treatment sites much thicker than 20 cm the new method results also in acceptable agreement with TPS calculations.

### E. *In vivo* dose verifications

*In vivo* EPID dose measurements were analyzed for ten breast cancer treatments with wedged 6 MV beams. These wedged beam treatments were all irradiations of the boost volume, which followed whole breast irradiation using an IMRT technique. Most of these wedged fields were not tangential but often orthogonal to the boost volume. The thickness of the breast, measured along the beam axis through the isocenter, varied for these ten patients between 11.8 and 22.3 cm and was on average 16.0±3.2 cm. The depth of the dose reconstruction point varied between 2.5 and 7.5 cm and was on average 4.0±1.6 cm.

The planned dose data in the plane perpendicular to the beam through the isocenter were compared with the dose values reconstructed from the corrected EPID images. The TPS dose distribution was calculated using a grid voxel size of 4 mm in all dimensions. The *in vivo* EPID‐based reconstructed dose values were corrected by applying the appropriate partial wedge correction factors and the resulting dose values were compared with the corresponding planned dose data. Details of the verified breast irradiations are shown in Table 1. In this part of the study the actual clinical approach was followed, which consists of verifying, *in vivo*, dose distributions in isocenter planes, or planes through the dose prescription point, perpendicular to the beam direction, using a 2D γ‐evaluation method (global 3%/3 mm criteria) within the 20% isodose line. The mean $\gamma \;(\gamma_{\text{mean}})$ and the 99th percentile of the γ distribution ($\gamma_{1\%}$, the near maximum γ), as well as the percentage of points with $\gamma \leq 1\;(P_{\gamma \leq 1})$, were included in the analysis. In addition the dose difference at the isocenter or prescription point is reported. For the analysis of the data obtained with the wedged beam model, the same four evaluation parameters were adopted. If the total dose at the isocenter is deviating more than 3.0%, a warning is given (tolerance level exceeded), while for a deviation larger than 5%, an error report is sent immediately to a physicist (action level exceeded). These alert criteria are the same for the situation with and without wedge.

**Table 1:** Overview of the 6 MV wedged beam breast cancer treatments used for the clinical verification of our method.

*Patient Number*	*Number Of Usable Wedged Beams*	*Number Of Corresponding EPID Images*
1	1	3
2	1	2
3	1	3
4	1	4
5	2	4
6	1	2
7	1	3
8	2	6
9	2	6
10	1	2

## III. RESULTS

### A. Wedge multiplication factors and wedge correction factors

The results of the EPID and ionization chamber measurements with and without a 20 cm thick polystyrene slab phantom in the beam were used for the determination of the wedge multiplication factors. The resulting wm*f* values are given in Table 2 for the three beam energies for the two linacs. The standard deviations were determined from an average over four separate measurements, two for both collimator angles (90° and 270°). It is assumed that the precision of the nonwedged IC measurements is equal to the precision of the wedged IC measurements when averaged over the two collimator angles.

**Table 2:** Wedge multiplication factors, wm*f*, and standard deviations (in brackets), derived from EPID and ionization chamber measurements performed at two linacs with and without a 20 cm thick polystyrene slab phantom in the beam, for three photon beam energies.

	*wmf Without the Phantom in the Beam*			*wmf With the Phantom in the Beam*		
*Linac*	*6 MV*	*10 MV*	*18 MV*	*6 MV*	*10 MV*	*18 MV*
A4	1.133	1.028	1.038	1.063	1.030	1.043
	(0.014)	(0.012)	(0.012)	(0.017)	(0.015)	(0.014)
B1	1.137	1.021	1.038	1.052	1.028	1.047
	(0.015)	(0.012)	(0.012)	(0.020)	(0.016)	(0.016)
Average	1.135	1.025	1.038	1.058	1.029	1.045
	(0.010)	(0.009)	(0.009)	(0.013)	(0.011)	(0.011)

Table 3 provides values for the wedge correction factors for the three different beam energies at 10 cm depth in the 20 cm thick slab phantom. The data in the first row are wc*f* values obtained by using the two‐factor model and the wm*f* data presented in Table 2. The standard deviations were derived by propagating the standard deviations given in Table 2 to our formula for the primary dose in the midplane. It is assumed here that the total midplane dose has a similar precision as the primary midplane dose. The second row gives the primary midplane dose correction calculated by means of Eq. (7), while the third row displays the total isocenter dose correction at the same position, but determined now as the ratio of the dose values calculated by the TPS and the dose reconstructed from uncorrected EPID images in 6, 10, and 18 MV wedged photon beams within a sphere with a radius 0.5 cm around the isocenter. These values were derived by combining the data from the two linacs. The small standard deviations in these data are caused by the small differences between corresponding data for the two accelerators. The three sets of wedge correction factors are in good agreement.

**Table 3:** Single wedge correction factors, wc*f*, for EPID‐based reconstructed dose distributions at 10 cm depth in a 20 cm thick slab phantom for the three photon beam energies. The first row gives the ratio between reconstructed dose values with and without corrections to the portal images obtained during wedged beam irradiations derived using the two‐factor model and the wm*f* data presented in Table 2. The second row shows values obtained using the approximate relationship between wc*f* and wm*f* values using the average data presented in Table 2. The third row shows the ratio of a direct comparison of the dose values determined by the TPS and the reconstructed dose values without corrections to the portal images. The dose values have been averaged over the volume of a sphere with a radius of 0.5 cm centered at the isocenter.

*Energy*	*6 MV*	*10 MV*	*18 MV*
two‐factor model	1.109	1.026	1.041
	(0.017)	(0.014)	(0.014)
$\surd wmf^{ph}.\surd wmf^{air}$	1.096	1.027	1.041
	(0.017)	(0.014)	(0.014)
comparison with TPS	1.116	1.024	1.037
	(0.012)	(0.016)	(0.001)

### B. Phantom dose verifications

**Figure 1:**
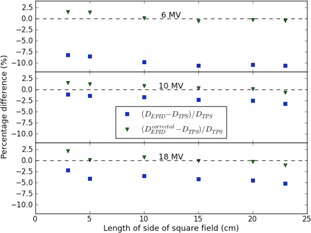
Percentage differences between EPID‐based dose values from square wedged fields at 10 cm depth in the middle of an isocentrically aligned 20 cm thick phantom, determined by the nonwedged ($D_{\text{EPID}}$) and wedged beam approach ($D_{\text{EPID}}^{\text{corrected}}$) and the dose values calculated by the TPS ($D_{\text{TPS}}$), as a function of field size for 6, 10, and 18 MV photon beams. Averaging has been done over a sphere with a radius of 0.5 cm around the isocenter. The data have been determined under isocentric conditions for 200 MUs. The zero levels for the percentage differences have been indicated by dashed lines.

The dose values determined by the EPID with and without the multiplication of the pixel values in the portal images (with the average wm*f* values from Table 2) were compared with the dose values determined by the TPS for the three energies as a function of field size, at 10 cm depth in the 20 cm thick polystyrene slab phantom at one of the linacs. EPID dose reconstructions from two sets of measurements were averaged. Figure 1 depicts the relative differences between EPID‐based reconstructed and planned dose values for 6, 10, and 18 MV, respectively.

This figure shows that the dose values determined by the EPID using the wm*f* values given in Table 2 are in good agreement with the data predicted by the TPS. The largest remaining difference is seen at 18 MV for a field size of $3\times 3\;{\text{cm}}^{2}$ (2.1% overdosage with respect to the TPS value).

Depth‐dose curves inside a phantom were determined in order to assess the improvement for depths other than 10 cm by correcting the portal images from wedged beams.

**Figure 2:**
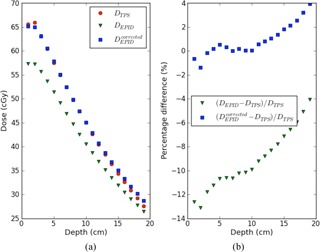
EPID‐based reconstructed depth dose curves (a) using the nonwedged ($D_{\text{EPID}}$) and wedged beam model ($D_{\text{EPID}}^{\text{corrected}}$), for a $10\times 10\;{\text{cm}}^{2}$ 6 MV wedged beam along the central beam axis in a 20 cm thick slab phantom for 200 MUs, compared with the data from the TPS ($D_{\text{TPS}}$). Percentage difference (b) between reconstructed and planned dose values as a function of depth for the same set of data.

Figure 2(a) shows depth‐dose curves for a 6 MV $10\times 10\;{\text{cm}}^{2}$ field, along the central beam axis in the 20 cm thick slab phantom for TPS dose values and for EPID‐based dose reconstructions with and without portal image corrections. Figure 2(b) presents the corresponding percentage differences between dose values (EPID‐TPS) / TPS for a 6 MV $10\times 10\;{\text{cm}}^{2}$ field. From these figures it can be seen that, by multiplying the pixel values in the portal images with the wm*f* data given in Table 2, the agreement between the TPS and EPID‐based depth‐dose curves improves considerably at all depths.

**Figure 3:**
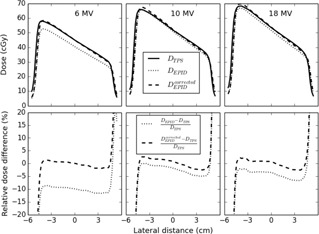
(Upper panel) Beam profiles along the wedge gradient determined by EPID dose reconstruction using the nonwedged ($D_{\text{EPID}}$) and wedged beam model ($D_{\text{EPID}}^{\text{corrected}}$) compared with the data from the TPS ($D_{\text{TPS}}$) for 6, 10, and 18 MV for a $10\times 10\;{\text{cm}}^{2}$ field at 10 cm depth in a 20 cm thick phantom for 200 MUs. (Lower panel) Percentage difference between reconstructed and planned dose values for the same set of data.

In order to verify the dose in the off‐axis region, beam profiles along the wedge gradient were taken from an EPID‐based dose reconstruction for a field size of $10\times 10\;{\text{cm}}^{2}$ at 10 cm depth in the slab phantom, with and without correction of the portal images using the wm*f* values from Table 2, and compared with the profiles predicted by our TPS. The reconstructed beam profiles in the wedge direction are shown in the top panel of Fig. 3.

**Figure 4:**
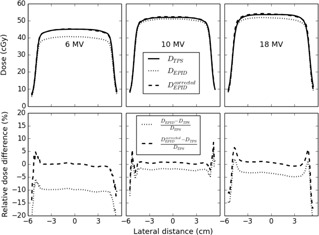
(Upper panel) Beam profiles perpendicular to the wedge gradient determined by EPID dose reconstruction using the nonwedged ($D_{\text{EPID}}$) and wedged beam model ($D_{\text{EPID}}^{\text{corrected}}$) compared with the data from the TPS ($D_{\text{TPS}}$) for 6, 10, and 18 MV for a $10\times 10\;{\text{cm}}^{2}$ field at 10 cm depth in a 20 cm thick phantom for 200 MUs. (Lower panel) Percentage difference between reconstructed and planned dose values for the same set of data.

For 6 MV the disagreement between the EPID and TPS profiles is clear, while the disagreement is much smaller for the other energies. The agreement for 6 MV is greatly improved in the high‐dose region when the wm*f* values are applied, while minor improvements are observed in the low‐dose region. The comparison between EPID‐based and TPS dose profiles in the direction perpendicular to the wedge gradient is shown in Fig. 4. Also in this direction better agreement can be seen for all energies when applying the wm*f* values and the improvement is again the largest for 6 MV.

### C. *In vivo* dose verifications

**Figure 5:**
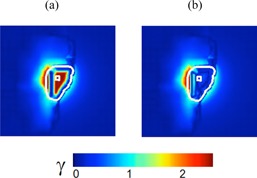
γ‐evaluation data in a plane through the dose specification point (small white square) showing: (a) verification for the fully wedged 6 MV beam of Patient 3 using the nonwedged beam approach; (b) verification with the same portal image using the wedged beam approach. The dimension of both images is 25.6 cm×25.6 cm. The white line indicates the intersection of the 20% isodose surface with the plane through the dose specification point perpendicular to the beam.

*In vivo* dose verification was performed for 13 (partially) wedged 6 MV beams for 10 breast cancer treatments. Table 4 shows the results of the 2D γ‐evaluation including the dose difference at the isocenter or dose prescription point, for the dose reconstruction with and without applying the new wedge model. It can be observed that a considerable improvement in the results for all but one beam is obtained when our correction procedure proposed for wedged beam irradiations is used. For only one beam with a relatively small $\text{wc}f^{\text{partial}}$ value (beam 1 of Patient 5) the results are slightly worse. The improvement is most prominent for the fully wedged beam applied to Patient 3, which is illustrated by the corresponding change in gamma values (see Fig. 5).

**Table 4:** Results for the dose at the isocenter/dose specification point and the γ‐evaluation for the *in vivo* verification of ten breast cancer treatments with 6 MV photon beams using the nonwedged and wedged beam approach.

	*Nonwedged Beam Approach*				*Wedged Beam Approach*				
*Patient Number*	$\Delta D_{isoc}$ *(EPID‐plan)*	$P_{\gamma \leq 1}$%	$\gamma_{mean}$	$\gamma_{1\%}$	${\mathrm{wcf}}_{\mathrm{partial}}$	$\Delta D_{isoc}$ *(EPID‐plan)*	$P_{\gamma \leq 1}$%	$\gamma_{mean}$	$\gamma_{1\%}$
1	−3.0%	70.2	0.69	1.8	1.024	−0.7%	97.2	0.34	1.1
2	−5.2%	52.8	1.0	2.2	1.038	−1.6%	95.0	0.47	1.2
3	−10.9%	49.0	1.4	3.3	1.109	−1.4%	85.0	0.59	3.0
4	−2.5%	72.5	0.72	1.6	1.024	−0.1%	97.5	0.38	1.2
5	0.2%	94.5	0.34	1.4	1.024	2.6%	94.5	0.47	1.1
5	−7.0%	55.7	1.0	2.4	1.061	−1.1%	85.9	0.51	2.2
6	−3.1%	79.3	0.57	1.7	1.024	−0.8%	98.0	0.32	1.1
7	−2.2%	87.1	0.57	2.0	1.021	−0.2%	93.1	0.40	1.5
8	−5.4%	51.9	1.16	2.7	1.068	1.1%	86.3	0.50	1.9
8	−5.3%	48.7	1.26	2.8	1.069	1.2%	85.1	0.57	2.3
9	−3.7%	64.1	0.86	2.1	1.056	1.7%	97.5	0.38	1.1
9	−3.5%	64.3	0.84	2.2	1.051	1.5%	76.9	0.69	2.6
10	−2.5%	62.7	1.16	5.6	1.046	2.0%	63.9	1.27	7.2
Weighted mean (1 SD)^a^	−5.3% (3.3)	60.2 (13.5)	1.01 (0.30)	2.6 (0.9)	1.061	0.4% (1.7)	87.4 (8.9)	0.55 (0.21)	2.3 (1.4)

^a^ The weighted mean has been calculated by weighting the EPID dose reconstructions from the individual portal images with a weighting factor equal to (${\mathrm{wcf}}_{\mathrm{partial}}$ −1).

It is instructive to average the results from the EPID dose reconstructions from all individual portal images corresponding to these 13 beams to assess the overall effect of our method. The results from the dose reconstructions were weighted with $\left(\text{wc}f_{\text{partial}}-1\right)$ in order to enhance the differences for beams having the largest wedge correction. The overall $\text{wc}f_{\text{partial}}$ equals 1.061 and improves the EPID reconstructed isocenter underdosage with respect to the Pinnacle data from 5.3%±3.3% to −0.4%±1.7% . Also, the improvements of $\gamma_{\text{mean}}$ from 1.01±0.30 to 0.55±0.21, and $P_{\gamma \leq 1}$ from 60.2%±13.5% to 87.4%±8.9% are compelling.

## IV. DISCUSSION

aSi EPID‐based dosimetry models, using a nonwedged beam pixel‐to‐dose conversion, present shortcomings in wedged beams, mainly for the lower photon beam energies. The aim of this work was to establish a practical wedged beam approach as generic as possible, maintaining the simple characteristics of an algorithm using an open‐beam calibration. For this purpose, two portal image correction factors for wedged beams (wedge multiplication factors) were introduced, for the situation with and without a phantom in the beam, each valid for a specific energy. This required two additional ionization chamber and EPID measurements, with and without a wedge in the beam, for both situations. These wedge multiplication factors can in principle be applied in any aSi EPID dosimetry model to convert EPID pixel values into dose values when applying nonwedged beams for calibration. Dose reconstruction in a patient/phantom using portal images corrected in this way showed, for the model applied in our center, significant improvements with respect to the uncorrected dose reconstructions, especially for 6 MV, without the need to have an additional calibration procedure for wedged beams.

The proposed new wedged beam model assumes that these two factors are only dependent on beam energy and applicable for all patient/phantom thicknesses, depths, field sizes, and off‐axis distances of clinical interest. This assumption was verified for our model for a number of field sizes, which demonstrated that the EPID‐based dose values using the wedged beam correction showed indeed good agreement with the TPS dose values for the three beam energies at 10 cm depth in the middle of a 20 cm thick phantom (see Fig. 1). Also the depth‐dose curves reconstructed from EPID measurements and calculated by the TPS in a 20 cm thick phantom for a $10\times 10\;{\text{cm}}^{2}$ 6 MV beam showed good agreement — within 2% for depths between dose maximum and about 15 cm depth (see Fig. 2(b)). This conformity is slightly worse compared to the accuracy of the depth‐dose reconstruction for a nonwedged $10\times 10\;{\text{cm}}^{2}$ 6 MV beam. For the $10\times 10\;{\text{cm}}^{2}$ fields, the lateral dose profiles were also compared for the three beam energies at 10 cm depth in a 20 cm thick phantom and showed good agreement with the planned dose values (within about 2%). It therefore seems reasonable to assume that the wedge multiplication factors measured for $10\times 10\;{\text{cm}}^{2}$ wedged beams and a 20 cm thick polystyrene slab phantom are applicable for a large range of clinically relevant situations.

A potential source of error in the application of the method described in this paper may exist from using a set of model parameters developed for one phantom size on an anatomical region of a patient having a different size. For breast cancer treatments using wedged beams, the results are good as can be deduced from the data presented in Table 4. However, the values of wm*f* as given in Table 2 still have to be determined for much thicker phantom/patient thicknesses in order to assess the limitation of the new method in applying these data for other treatment sites irradiated with wedged beams.

Wedge multiplication factors can also be applied in other models to convert aSi EPID pixel values into dose values when applying nonwedged beams for calibration. In pretreatment dose verification models, comparing measured portal dose images with those predicted by a TPS, as for instance those developed by Van Esch et al.^(13)^ and Nicolini et al.,^(14)^ multiplying the EPID image measured in a wedged beam with a $\text{wm}f^{\text{air}}$ factor might give good agreement between measured and calculated dose values. The same procedure, multiplying the EPID image but now with $\text{wm}f^{\text{ph}}$, can be applied for transit dosimetry models using EPID images measured behind phantom/patients (e.g., the models developed by François et al.^(17)^ and Berry et al.^(18)^) It would be interesting to verify if such a simple approach of using a wedge multiplication factor would indeed be sufficient to get accurate dose verification for wedged beams using these models.

The data given in Table 2 show that the wm*f* value for the 6 MV beam is smaller with the phantom in the beam. This is due to the beam hardening already caused by the phantom, so the beam hardening effect of the wedge decreases. For the other two higher energy beams, there is within the experimental uncertainty no difference between the two sets of data. Detailed Monte Carlo calculations are needed to explain quantitatively the effect of placing a phantom and a wedge in the beam on the EPID response for the three photon beam energies, for instance to clarify why the wm*f* values for 10 MV are lower than for 18 MV. The data presented in Table 2 also show that the differences between the wm*f* values for the situation with and without a phantom in the beam are small for 10 and 18 MV. Consequently, the transmission of the primary dose through the patient/phantom will hardly change when a wedge is inserted in these higher energy beams. EPID dosimetry models based on transmission, such as the one used by Berry et al.^(18)^ or the one used in our institution, therefore need for higher energies only a correction with $\text{wm}f^{\text{ph}}$ of the EPID image measured behind a patient/phantom. Another result of this observation is that, when applying in a back‐projection model a calculated value for the transmission instead of a measured value, as proposed by Pecharroman‐Gallego et al.,^(22)^ no additional correction is needed for higher energy wedged beams. However for 6 MV and probably also at lower photon beam energies, the calculated transmission has to be modified with the ratio of the two wm*f* values obtained from measurements with and without a phantom in the beam.

In case a single‐factor approach is used for the situation when only the summed EPID image of the irradiations with and without wedge in the beam are available, then $\text{wc}f_{\text{partial}}$ has to be determined using Eq. (6), which requires knowledge of wc*f*. As can be seen from Table 3, the wc*f* values obtained by applying the two‐factor model, the approximate formula results, and the values obtained by comparison with the TPS, are in good agreement. The small difference for the 6 MV beam between the wc*f* values obtained with the two‐factor model and the square root of the two wm*f* values is caused by an approximation in the scatter contribution to the dose involved in deriving Eq. (7). It should be noted that the single‐factor approach will probably give good results under a number of circumstances, but may do worse at depths further away from the midplane. The two‐factor approach is more universal and valid for a range of depths, and might therefore give more accurate results for more extreme patient thicknesses and field sizes, but requires ionization chamber measurements.

*In vivo* dose verification of wedged beam treatments using the EPID‐based dosimetry model as applied in our center, in combination with the proposed wedged beam corrections, demonstrated good results both for the dose at the isocenter/dose prescription point and for the γ‐evaluation. This is in agreement with the data presented in Fig. 1 showing that, for the depths of the dose prescription point in these patients (i.e., between 2.5 and 7.5 cm), the difference between the reconstructed and predicted dose values is less than 1% when using the new wedged beam approach. The results shown in Table 4 confirm that an EPID‐based back‐projection model using the wedged beam approach can be used to accurately verify treatment plans that apply wedges even for the most unfavorable situation (i.e., in fully wedged low‐energy beams). A more extensive clinical validation of our wedged beam model was, however, compromised by the lack of EPID images. In our center, *in vivo* EPID dose verification was introduced simultaneously with IMRT, while IMRT gradually rendered wedged beams redundant. In other words, *in vivo* EPID dosimetry and the use of wedges were mutually exclusive in clinical practice. For that reason we could only reanalyze a small number of wedged beam irradiations. Also, wc*f* and $\text{wc}f_{\text{partial}}$ values are much smaller for wedged 10 and 18 MV beams than for 6 MV beams. Hence it would require averaging over many patient measurements to reach the statistical significance necessary to surpass the overall uncertainty of EPID‐based dose reconstructions for these higher energy beams. Since these data were not available, only 6 MV treatments were investigated.

## V. CONCLUSIONS

The multiplication of pixel values in portal images with two energy‐dependent correction factors, derived from EPID and ionization chamber measurements, to take the change in photon energy spectrum by insertion of a wedge in the beam into account, provides good agreement between dose values determined by an aSi EPID and a TPS. The results of these phantom measurements, as well as those of *in vivo* dose verifications in wedged beams using the new model, indicate that such a practical approach, without an extra commissioning effort, may also be used in other models to determine accurately the dose distribution with aSi EPIDs in a number of clinical situations of treatments with wedged beams.
